# Development of a combined RLEP/16S rRNA (RT) qPCR assay for the detection of viable M. leprae from nasal swab samples

**DOI:** 10.1186/s12879-019-4349-9

**Published:** 2019-08-28

**Authors:** Marcus Beissner, Anna Woestemeier, Malkin Saar, Kossi Badziklou, Issaka Maman, Charlotte Amedifou, Magdalena Wagner, Franz X. Wiedemann, Komi Amekuse, Basile Kobara, Karl-Heinz Herbinger, Abiba Banla Kere, Thomas Löscher, Gisela Bretzel

**Affiliations:** 1 Department of Infectious Diseases and Tropical Medicine (DITM), Medical Center of the University of Munich (LMU), Munich, Germany; 2 Ministère de la Santé, Institut National d’Hygiène (INH), Lomé, Togo; 3 German Leprosy and Tuberculosis Relief Association, Togo office (DAHW-T), Lomé, Togo; 4 Programme National de Lutte contre l’Ulcère de Buruli, la Lèpre et le Pian (PNLUB-LP), Lomé, Togo; 5 Ludwig-Maximilians-University (LMU), Munich, Germany

**Keywords:** *Mycobacterium leprae*, RLEP qPCR, Bacillary load, 16 rRNA RT qPCR, Viability, Nasal swab samples

## Abstract

**Background:**

Leprosy continues to be a health problem in endemic areas. More than 200,000 new cases of leprosy per year suggest that transmission of the disease is still ongoing, presumably as airborne infection through nasal droplets. Late diagnosis supports continued transmission and increases the individual risk for functional disabilities. Laboratory tools are considered beneficial to facilitate early detection and clinical assessment of cases. The aim of this study was to validate molecular tools allowing detection, quantification and assessment of viability of *M. leprae* from nasal swab samples which are easy to obtain without the need of any invasive procedures.

**Methods:**

Validation of two real-time PCRs detecting *M. leprae* DNA (RLEP qPCR) and RNA (16S rRNA RT qPCR) was conducted on “must not detect”/“must detect” samples and 160 pre-treatment nasal swab samples from 20 clinically diagnosed multibacillary (MB) leprosy patients from Togo.

**Results:**

Both assays were 100% *M. leprae* specific and showed analytical sensitivities of three templates each. Out of 20 clinically diagnosed MB leprosy patients, 15 (75.0%) had a positive RLEP qPCR result from nasal swab samples. The 16S rRNA RT qPCR detected viable bacilli in nasal swab samples of ten out of these 15 RLEP positive patients (66.7%).

**Conclusion:**

The combined RLEP/16S rRNA (RT) qPCR assay provides a sensitive and specific tool to determine the bacterial load and viability of *M. leprae* from nasal swab samples and is applicable for early diagnosis, monitoring treatment response and investigating the role of nasal carriage of *M. leprae* in human-to-human transmission through aerosol infection.

**Electronic supplementary material:**

The online version of this article (10.1186/s12879-019-4349-9) contains supplementary material, which is available to authorized users.

## Background

Leprosy caused by *Mycobacterium leprae* is a neglected, chronic infectious disease predominantly affecting skin and peripheral nerves. More than 200,000 new cases are detected annually and up to 60% of patients have peripheral nerve damage at diagnosis and are therefore prone to long-term morbidity. Environmental sources may be involved in the ongoing dissemination of *M. leprae*, but aerosol spread through the upper respiratory tract is considered the principal means of transmission. The disease is spectral and categorized according to the Ridley & Jopling classification [1]. Patients with a strong cell-mediated immune response have few lesions with few or no detectable mycobacteria (tuberculoid forms), whereas patients anergic to *M. leprae* have multiple lesions with numerous mycobacteria (lepromatous forms). Between these two poles borderline forms exist. To guide treatment decisions, WHO has introduced a simplified classification based on the number of lesions. Whereas previously paucibacillary (PB) cases (up to five skin lesions) were treated for six months with rifampicin and dapsone, and multibacillary (MB) cases (more than five skin lesions) for 12 months with rifampicin, dapsone and [2] clofazimine, current WHO recommendations valid since August 2018 envisage to treat PB patients for six months and MB patients for 12 months with rifampicin, dapsone and clofazimine (multi-drug therapy, MDT). The diagnosis of leprosy is clinical. However, an estimated 30% of patients, including many MB cases, do not present conclusive clinical signs and classification of patients based on counting of lesions alone is subject to error. Therefore, application of auxiliary laboratory based tools is considered beneficial to support clinical diagnosis and classification. Well-established procedures are histological diagnosis as well as determination of the bacteriological index (BI, representing the quantitative bacillary load) and the morphological index (MI, representing the percentage of intact solid stained and presumably viable bacilli) by means of examination of Ziehl-Neelsen stained slit skin smears (SSS). Furthermore, phenolic glycolipid I serology is considered an excellent surrogate marker for the bacterial load and can aid in clinical management such as patient classification and monitoring of treatment [3–10].

Considerable progress has been made in the field of molecular diagnostics. PCR techniques have been applied to investigate possible environmental sources for dissemination of *M. leprae* as well as the aerosol route of infection by means of nasal carriage [11–13]. Regarding diagnostics, quantitative polymerase chain reaction (qPCR) technology is considered at least 20 times more sensitive than microscopic detection and becomes increasingly important for early diagnosis and for difficult-to-diagnose cases, such as patients with negative microscopy, pure neural leprosy, or differential diagnosis of lesions with inconclusive histopathology [14]. Although the diagnostic sensitivity of (q) PCR assays is considered highest for skin biopsies, *M. leprae* DNA detection rates of more than 80% from SSS and nasal swab samples from clinically suspected MB cases, as well as 30–40% from SSS and nasal samples of BI negative PB cases were reported. Among a range of possible gene targets, the *M. leprae* specific repetitive element RLEP with an amplifiable copy number varying between 19 and 37 according to mutations in the primer binding sites, has been identified as the most suitable target for diagnostic applications [14–20].

As diagnostic PCRs only amplify *M. leprae* DNA and the pathogen cannot be cultured in-vitro, alternative technologies are required to determine mycobacterial viability. Molecular viability assays targeting ribosomal (e.g. 16S rRNA) or messenger RNAs - only detectable from viable/replicating bacteria – are available for monitoring treatment of patients with tuberculosis and Buruli ulcer. Comparable viability assays were developed for *M. leprae* and are applicable for environmental studies and assessment of treatment response in leprosy patients [11, 12, 14, 21–23].

This study describes the technical and clinical validation of a novel combined RLEP qPCR and 16S rRNA RT qPCR assay as a suitable method for detection, quantification and assessment of viability of *M. leprae* from nasal swab samples. To the best of our knowledge, it is the first application of this combined molecular diagnostic approach on samples which can easily be obtained without the need of invasive procedures.

## Methods

### Samples used for development and technical validation of RLEP qPCR and 16S rRNA RT qPCR

Technical validation of the combined assay was performed with “must detect RLEP/16S rRNA (DNA)” samples and “must not detect RLEP/16S rRNA (DNA)” samples as indicated in Table 1.

**Table 1:** Samples for technical validation of RLEP qPCR and 16S rRNA RT qPCR

Purpose	Sample type (No.)	Obtained from	Origin
“Must detect RLEP/16S rRNA (DNA)” samples	Nasal swab sample (4)^a^	2 sequencing confirmed MB leprosy patients	Togo
	PCR standard (1 per RLEP and 16S rRNA qPCR)	Cloned RLEP/16S rRNA plasmids with known copy numbers	GenExpress, Berlin, Germany
“Must not detect RLEP/16S rRNA (DNA)” samples	Nasal swab sample (14)^a^	7 endemic controls (healthy individuals)	Togo
	Nasal swab sample (10)^a^	5 occupational contacts to untreated (MB) leprosy patients	Munich, DITM^b^ medical staff
	Nasal swab sample (6)^a^	3 non-exposed healthy controls	Munich, DITM^b^ laboratory staff
	Swab sample (5)^a^	5 PCR confirmed Buruli ulcer patients	Ghana [24]
	Fine needle aspirate (12)^a^	11 PCR confirmed Buruli ulcer patients	Ghana [24]
	Swab sample (1)^a^	3 Patients with PCR confirmed cutaneaous leishmaniasis	Munich, accredited diagnostic laboratories of DITM^b^
	Punch biopsy sample (2)^a^		
	Mycobacterial culture (13)^a^	*M. abscessus, M. africanum, M. avium, M. bovis, M. fortuitum, M. gordonae, M. intracellulare, M. kansasii, M. malmoense, M. marinum, M. microti, M. tuberculosis, M. xenopi*	National Reference Center for Mycobacteria, Borstel, Germany
	Bacterial culture (5)^a^	Microbial flora colonizing human skin or nasal mucosa: *Propionibacterium acnes, Staphylococcus aureus, Staphylococcus epidermidis, Streptococcus pyogenes and Escherichia coli*	Max von Pettenkofer-Institute, Ludwig-Maximilians-University, Munich, Germany

^a^ DNA extracts (additional file – protocol 1)

^b^ DITM, Department of Infectious Diseases and Tropical Medicine (accredited according to DIN EN ISO 15189)

The “must detect RLEP/16S rRNA (DNA)” samples were derived from two clinical diagnosed MB leprosy patients from Togo. To confirm the presence of *M. leprae* DNA in the respective samples and to analyze the RLEP nucleotide sequence of Togolese *M. leprae* strains, a conventional PCR was designed for amplification of the *M. leprae* repeated element (RLEP) for direct DNA sequencing. Briefly, primers RL-F2 and RL-R2 were designed using Primer BLASTN (GenBank, NCBI) and DNAsis max 3.0 (MiraiBio Group, San Francisco, CA) by excluding significant concordances with human DNA and bacteria colonizing human skin or mucosae (Table 2).

**Table 2:** Sequences of applied primers and probes

Test	Primer/probe^a^	Sequence (5′- 3′)^b^	Nucleotide position^c^	Amplicon size^d^
RLEP PCR^e^	RL - F2	ACC TGA TGT TAT CCC TTG CAC	39,741–39,761	167 bp
	RL - R2	*CGC TAG AAG GTT GCC GTA TG*	39,908–39,889	
RLEP qPCR	RLEP - F	GCA GTA TCG TGT TAG TGA A	39,839–39,857	69 bp
	RLEP - R	*CGC TAG AAG GTT GCC GTA TG*	39,908–39,889	
	RLEP - P	6FAM- *CGC CGA CGG CCG GAT CAT CGA* -BBQ	39,885–39,865	
16S rRNA RT qPCR	ML16S rRNA TaqF	GCA TGT CTT GTG GTG GAA AGC	1,341,385–1,341,405	70 bp
	ML16S rRNA TaqR	*CAC CCC ACC AAC AAG CTG AT*	1,341,455–1,341,436	
	ML 16S - TP2	6FAM- *CCA TCC TGC ACC GCA AAA A* -BBQ	1,341,424–1,341,406	
GAPDH^f^ (RT) qPCR	GAPDH fwd	GAA GGT GAA GGT CGG AGT C	194–212	225 bp
	GAPDH rev	*GAA GAT GGT GAT GGG ATT TC*	419–400	
	GAPDH TM	FAM-*CAA GCT TCC CGT TCT CAG CCT* -BBQ	390–370	

^a^*F* Forward primer, *R* Reverse primer, *P/TP/TM* Hydrolysis probes (TibMolBiol, Berlin, Germany)

^b^ Hydrolysis probe with 6-Caboxyfluorescein fluorescent dye (6FAM) and BlackBerry Quencher (BBQ)

^c^ Nucleotide positions are provided for the first copy of the respective amplicon in *Mycobacterium leprae* Br4923 (GenBank accession number FM211192.1). For GAPDH qPCR nucleotide positions are provided for the copy in *Homo sapiens* glyceraldehyde-3-phosphate dehydrogenase (GenBank accession number NM_002046.5) [25]

^d^ bp = base pairs

^e^ Direct DNA sequencing was conducted with the forward primer RL-F2. The sequence encompassed the region amplified by RLEP qPCR

^f^ GAPDH = glyceraldehyde-3-phosphate-dehydrogenase

PCR amplification and purification of PCR products was conducted as described in the Additional file 1: Protocol 2 followed by direct DNA sequencing using primer RL-F2 as previously described [24–26].

### Primers, probes and PCR protocols

For amplification and detection of RLEP (*M. leprae* DNA), oligonucleotide sequences for primers and the hydrolysis probe designed by Truman et al. were used [27]. By means of RLEP sequencing significant mutations in the RLEP regions targeted by primers and the hydrolysis probe were excluded for the Togolese *M. leprae* strains. Therefore, a minimum of 19 RLEP copies were expected to be amplified per *M. leprae* genome. The assay was optimized for application on a CFX96 real-time PCR detection system (BioRad, Munich, Germany; as used by the Department of Infectious Diseases and Tropical Medicine [DITM], Munich, Germany) and ABI PRISM 3100 Genetic Analyzer (Applied Biosystems, Foster City, California; as used by the “Institut National d’Hygiène” [INH], Lomé, Togo). Furthermore, stability of reagents at ambient temperature (20–35 °C) was considered in view of intercontinental shipment. Modifications included the fluorescent dye (6-Carboxyfluorescein [6-FAM]) and quencher (BlackBerry quencher [BBQ]) of the hydrolysis probe (Table 2) (Tib MolBiol, Berlin, Germany), the qPCR mix (5x HOT FIREPol Probe qPCR Mix Plus, Solis ByoDyne, Tartu, Estonia), the exogenous internal positive control (Life Technologies, Darmstadt, Germany) and the qPCR run protocol (Additional file 1: Protocol 3).

For amplification and detection of the *M. leprae* 16S ribosomal RNA (rRNA) gene, primers published by Martinez et al. [22] were used in combination with a hydrolysis probe (TibMolBiol) modified with 6-FAM and BBQ for thermodynamic reasons (Table 2). Like for RLEP qPCR, modifications of the reagents and run protocol of the 16S rRNA qPCR were employed (Additional file 1: Protocol 4).

Controls included in each qPCR are summarized in Table 3.

**Table 3:** Controls for RLEP qPCR and 16S rRNA RT qPCR assays

Control	Purpose	Material/ method	
		RLEP qPCR	16S rRNA RT qPCR
Negative extraction control	To exclude contamination during extraction procedure	Transport buffer extracted in the same way as samples	NA^a^
Positive run control	To ensure adequate performance of qPCR	Cloned RLEP plasmid standard	Cloned 16S rRNA plasmid standard
Negative no template control	To exclude contamination during PCR set up	DEPC^a^ treated water	DEPC^b^ treated water
Internal positive control	To exclude false negative results due to inhibition	TaqMan exogenous internal positive control (IPC)^c^	TaqMan exogenous internal positive control (IPC)^c^

^a^*NA* Not applicable

^b^*DEPC* Diethylpyrocarbonate

^c^Applied Biosystems, Frankfurt, Germany

### Determination of RLEP copy numbers and bacillary loads

In absence of genomic data on Togolese *M. leprae* strains, the definition of analytical sensitivity as limit of detection (LOD; lowest template concentration rendering positive amplification of 95% of samples) of the RLEP qPCR required determination of RLEP copy numbers (copy number variation assay [CNV]). In brief, exact quantification of 16S rRNA gene (DNA) and RLEP element numbers was conducted by 16S rRNA qPCR and RLEP qPCR using logarithmic dilutions of plasmid standards (GenExpress, Berlin, Germany) to establish a standard curve. As the number of 16S rRNA genes (occurring in one copy per genome [GenBank, PubMed, NCBI]) corresponds to the amount of *M. leprae* bacilli per whole genome extract, the number of RLEP copies was calculated by dividing the number of RLEP elements by the number of 16S rRNA genes. Thus, the mean RLEP copy number (CN) per Togolese *M. leprae* genome was obtained for exact quantification of *M. leprae* genomes in clinical samples by RLEP qPCR. Exact quantification was conducted by the standard curve method using serial dilutions (10^7^–10^3^) of plasmid standards separately prepared for each run and used within 24 h. The RLEP copy number per template was provided as “starting quantity” (SQ) by the BioRad CFX99 based on the cross threshold (Ct) value, the slope of the regression line (y) and the crossing point of the standard curve with the Y-axis (b) with SQ = 10^(Ct − b)/y^. The bacillary load (BL) of samples was calculated by BL = (SQ x [volume of DNA extract/volume of template])/CN.

### Performance characteristics of RLEP qPCR and 16S rRNA RT qPCR

Specificity of the assays was assessed in silico using the basic local alignment search tool (BLAST, GenBank, NCBI) [25] and in vitro by testing the above mentioned “must not detect RLEP/16S rRNA (DNA)” samples. The LOD was determined by using 10-fold serial dilutions of cloned RLEP or 16S rRNA plasmid standards [28].

To assess qPCR efficiency, a standard curve was generated by means of 6 logarithmic dilutions of the plasmid standards which were subjected to the assays in quadruplicate. The efficiency (E) was calculated using the slope of the regression line (y) of the standard curve with E = 10^–1/y^-1. E values ≥ 0.95 were defined acceptable. In accordance with MIQE guidelines, the intra-assay variability was evaluated by testing each sample from the respective logarithmic dilution in triplicate within one 96-well qPCR plate in one run. Inter-assay variability was assessed by testing each sample on three subsequent days [28]. Variability was judged low if the maximum cycle threshold variation range (Ct-range_max_; i.e. range of Ct-values of samples tested in the same dilution) was ≤ 0.5 (intra-assay) and ≤ 1.0 (inter-assay). The maximum coefficient of variation of Ct-values (CV_max_, i.e. the ratio of the standard deviation to the mean of Ct-values from samples tested in the same dilution) was calculated to confirm significantly low variabilities with CV_max_-values ≤ 1.5 (intra-assay) and ≤ 3.0 (inter-assay).

### Clinical validation of RLEP qPCR and 16S rRNA RT qPCR

**Fig. 1:**
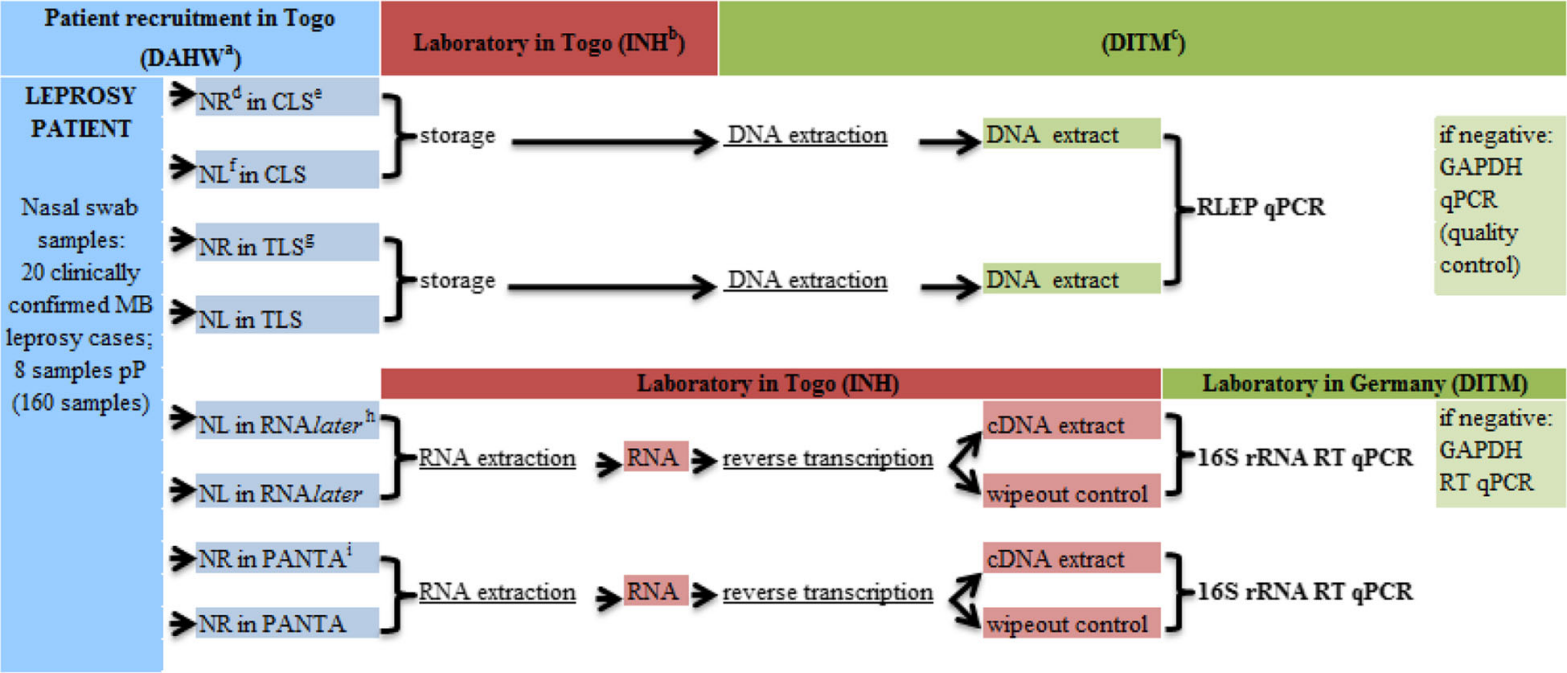
Overview of the sample processing during clinical validation. ^a^ DAHW = German Leprosy and Tuberculosis Relief association. ^b^*INH* Institut National d’Hygiène. ^c^*DITM* Department of Infectious Diseases & Tropical Medicine. ^d^ NR = nasal swab, right nostril. ^e^ *CLS* Cell lysis solution. ^f^*NL* Nasal swab, left nostril. ^g^*TLS* Tissue lysis buffer. ^h^ *RNAlater* RNA*later* stabilization reagent. ^i^*PANTA* PANTA transport medium

An overview of the complete sample processing is shown in Fig. 1.

### Study population and samples used for clinical validation

From May through October 2012, 20 clinically confirmed MB leprosy cases - detected in the context of the national leprosy control programme activities - were enrolled for clinical validation (inclusion criteria: > 5 years of age, > 5 skin lesions, no previous MDT). The cases originated from the regions Maritime (*n* = 12), Plateaux (*n* = 6), Centrale (*n* = 1) and Kara (*n* = 1).

Eight nasal swab samples per patient (*n* = 160) were collected with custom-made swabs (Bio-Budget, Krefeld, Germany). Two samples each (one per nostril) were stored in 700 μl cell lysis solution (CLS, Qiagen, Hilden, Germany) and 400 μl tissue lysis buffer (TLS, Bio-Budget) respectively for RLEP qPCR. Two other samples each (one per nostril) were stored in 500 μl PANTA transport medium (comprising **P**olymyxin B, **A**mphotericin B, **N**alidixic acid, **T**rimethoprim, **A**zlocillin; BD, Heidelberg, Germany, Additional file 1: Protocol 5) and 500 μl RNA*later* stabilization reagent (Qiagen) respectively for 16S rRNA RT qPCR.

### Extraction protocols, reverse transcription, sample storage and transport

Clinical samples were transported to the INH at ambient temperature in an upright position and within 48 h of collection. Samples for RLEP qPCR were stored at − 20 °C at INH until shipment to DITM by courier service and subsequently extracted by means of the Gentra Puregene method (Qiagen, Additional file 1: Protocol 1) for samples in CLS, or by means of a FastPrep extraction (Bio-Budget, Additional file 1: Protocol 6) for samples in TLS.

Samples for 16S rRNA RT qPCR were subjected to combined DNA/RNA extraction by means of the AllPrep DNA/RNA Micro Kit (Qiagen) at INH (Additional file 1: Protocol 5 & 7). Subsequently, whole transcriptome RNA was transcribed to complementary DNA by means of the QuantiTect Reverse Transcription Kit (Qiagen) with random hexamer primers, genomic DNA digestion and a gDNA wipe-out control (Additional file 1: Protocol 7) and samples were transported to DITM.

DNA and cDNA samples were subjected to RLEP qPCR and 16S rRNA RT qPCR (Additional file 1: Protocols 3 & 4).

### GAPDH (RT) qPCR

To exclude false negative RLEP qPCR or 16S rRNA RT qPCR results (e.g. DNA or RNA degradation during sample transport and/or extraction procedures) a glyceraldehyde-3-phosphate-dehydrogenase (GAPDH) mRNA (RT) qPCR was applied on all RLEP qPCR and 16S rRNA RT qPCR negative samples (excluding PANTA samples, as this medium does not preserve human RNA). The GAPDH mRNA (RT) qPCR detects human DNA and cDNA transcribed from mRNA.

Oligonucleotide sequences for primers were used as originally described by Janssens et al. [29]. The hydrolysis probe was modified with 6-FAM and BBQ for thermodynamic reasons (Table 2) and modifications of the reagents and run protocol were employed as described for *M. leprae* (RT) qPCR assays (Additional file 1: Protocol 8).

### Statistical analysis and comparative testing of clinical samples

All data was analyzed with Microsoft Excel and Stata (College Station, Texas, USA). An approximate test and estimation of standard error of proportion (SEP) to calculate one-sided 95% confidence intervals [95%-CI] of categorical test results were conducted. The 95%-CI of relative risk was calculated by means of method of Katz for the ratio of proportions (p): 95%-CI = p +/− z_1-α_ * SEP. The SEP was calculated by taking the root of (p * (1-p)/n) (*n* = number of statistical population observed). Significant differences were defined as non-overlapping 95%-CI of proportions.

## Results

### Technical and clinical validation of RLEP qPCR and 16S rRNA RT qPCR

#### Copy number variation assay

Analysis of five DNA extracts from four Togolese leprosy patients revealed a mean of 30 amplifiable RLEP copies per genome (standard deviation: 5.54) (Table 4).

**Table 4:** RLEP copy numbers according to CNV assay

Patient	Sample	Number of RLEP elements	Number of 16S rRNA genes	Amplified RLEP copy numbers per genome
1	Swab left nostril	23,443	928	25
	Swab right nostril	303	12	25
2	Swab left nostril	4778	12	36
3	Swab left nostril	31,405	1085	29
4	Swab left nostril	930	26	36

#### Specificity and analytical sensitivity

The assays were 100% *M. leprae* specific as all 68 “must not detect RLEP/16S rRNA (DNA)” samples tested negative. The LODs were three templates of the respective target sequence, i.e. 0.1 *M. leprae* genome equivalents for RLEP qPCR and three *M. leprae* genome equivalents for 16S rRNA RT qPCR.

#### Efficiency

**Fig. 2:**
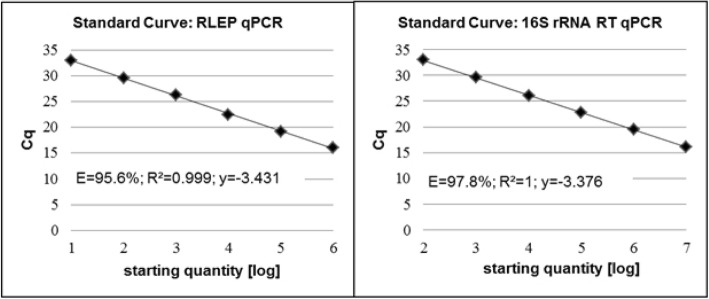
Standard curves for a) RLEP qPCR and b) 16S rRNA RT qPCR. Cq quantification cycle, *log* logarithmic, *E* Efficiency, *R*^*2*^ Coefficient of correlation; y = regression line

For RLEP qPCR the slope of the regression line was y = − 3.431 with a coefficient of correlation R^2^ > 0.99 and the efficiency (E) was 95.6%. For 16S rRNA RT qPCR the regression line was y = − 3.376 with a coefficient of correlation R^2^ > 0.99 and E = 97.8% (Fig. 2).

#### Intra- and inter-assay variability

Both qPCR assays revealed low intra- and inter-assay variabilities. RLEP qPCR revealed a Ct-range_max_ of 0.34 and a CV_max_ of 0.63 (intra-assay) as well as a Ct-range_max_ of 0.43 and CV_max_ of 1.50 (inter-assay). 16S rRNA RT qPCR revealed a Ct-range_max_ of 0.32 and a CV_max_ of 0.64 (intra-assay) as well as a Ct-range_max_ of 0.41 and CV_max_ of 1.17 (inter-assay) (Additional file 1 :Tables S1 & S2).

#### Clinical validation of RLEP qPCR and 16S rRNA RT qPCR

Out of 20 clinically diagnosed leprosy patients, 15 (75.0%; 95%-CI: 59.07; 90.93) had a positive RLEP qPCR result. Accurate sample collection, transport and DNA extraction was assured through 100% positive GAPDH qPCR results for all five RLEP qPCR negative patients.

Out of 15 RLEP qPCR positive patients, 10 (66.7%; 95%-CI: 46.64; 86.69) also tested positive by 16S rRNA RT qPCR. Among the remaining five 16S rRNA RT qPCR negative patients, GAPDH RT qPCR was positive for four (80.0%; 95%-CI: 44.94; 100) confirming the presence of human mRNA and therefore accurate sample collection, transport and whole transcriptome RNA extraction and cDNA synthesis.

#### Comparison of transport buffers

Comparison of transport buffers CLS and TLS revealed more positive RLEP qPCR results for samples extracted from CLS (28/40, 70.0%; 95%-CI: 58.08; 81.92) compared to TLS extracts (21/40, 52.5%; 95%-CI: 39.51; 65.49; not significant). Out of 28 RLEP qPCR positive CLS samples, 21 corresponding TLS samples also tested positive.

Comparison of transport buffers PANTA and RNA*later* showed slightly higher RNA detection rates by 16S rRNA RT qPCR from RNA*later* (13/40, 32.5.0%; 95%-CI: 20.32; 44.68) than from PANTA (10/40, 25.0%; 95%-CI: 13.74; 36.26; not significant). Out of 13 16S rRNA RT qPCR positive RNA*later* samples, eight corresponding PANTA samples also tested positive.

## Discussion

Mainly attributable to the introduction of MDT in the 1980s and its widespread free of charge distribution since 1995 through WHO, the leprosy elimination goal (i.e. < 1 patient per 10,000 population) was achieved in most countries in 2000. However, worldwide more than 200,000 new cases are still reported each year indicating ongoing transmission of the disease. In particular late diagnosis supports continued transmission and increases the individual risk for functional disabilities [7, 30]. Among the available diagnostic techniques applicable for laboratory confirmation, RLEP qPCR has shown the highest specificity and sensitivity for the detection of *M. leprae* in clinical samples. A range of studies validated the performance of RLEP qPCR on skin biopsies and SSS. Validation data for nasal swabs, a less invasive sampling technique which is applicable in the field, are however scarce. In addition, only a few studies applied an RLEP assay covering the complete range of the known RLEP variants, RLEP 1–4 [13, 14, 17, 20, 27, 31]. Therefore, the aim of this study was to validate an RLEP qPCR targeting the entire set of RLEP sequences applicable for nasal swabs.

Technical validation revealed a RLEP qPCR specificity of 100%, a very high analytical sensitivity of 0.1 *M. leprae* genome equivalents, an efficiency of 95.6% and low intra- and inter-assay variabilities. The RLEP qPCR positivity rate of 75% falls within the range of data reported from other studies applying RLEP qPCR for the detection of *M. leprae* DNA from nasal swab samples (47–75% [13, 31]), SSS (83% [20]) and skin biopsies (87% [17]) from clinically classified MB patients.

Beside early identification of clinical leprosy among RLEP qPCR positive contacts, one possible application of RLEP qPCR consists in monitoring the decrease of the bacillary load during MDT. However, *M. leprae* DNA is detectable for at least two years after MDT and mere quantification of bacilli does not provide definite information on the viability of possible remaining organisms. As *M. leprae* cannot be cultured on artificial media, proof of viability requires more sophisticated tools. Detection of *M. leprae* RNA is considered a suitable alternative to identify viable/replicating organisms, and a few studies demonstrated the applicability of RNA assays for assessment of bacterial viability under MDT in SSS and skin biopsies [14, 21, 22]. RNA assays have also gained an important role in transmission studies with viable *M. leprae* being detected in environmental samples derived from the immediate vicinity of houses of leprosy patients [11, 12]. To our knowledge application of RNA assays to investigate human-to-human transmission is still pending.

In our setting, a viability assay should serve two purposes. First, there was the need for a diagnostic tool to monitor the treatment response of leprosy patients attending our outpatient clinic in Munich, Germany. Furthermore, an assay facilitating transmission studies in research settings in Africa was required. As already shown by Martinez et al. analysis of *M. leprae* mRNA (e.g. *sod*A) to predict viability of the bacilli is limited to short-term experimental settings due to a low sensitivity in clinical samples [22]. The low sensitivity for mRNA detection from clinical samples in general was also described for other (myco-) bacterial pathogens [32], but some authors argued that rRNA – though highly sensitive – may also be detected from dead bacteria (among these also metabolic active but culture negative bacilli, e.g. MTBC) [32–34]. However, findings of rRNA analyses obtained from other pathogens may not be one-to-one transferred to *M. leprae* due to the genus- and even species-specific ribosomal transcriptome [35] and RNA applications for *M. leprae* should follow the existing knowledge on this specific species. For *M. leprae* Prakoeswa et al. recently proofed the findings of an earlier study by Haile et al. that 16S rRNA is rapidly degraded in dead *M. leprae* and can thus be used as marker of viability [36, 37]. Therefore, based on the 16S rRNA RT qPCR first described by Martinez et al. [22], a viability assay for non-invasive nasal swab samples - facilitating repeated sampling as well as sampling under field conditions - was designed. Technical validation revealed a high analytical sensitivity of three *M. leprae* genome equivalents, qPCR efficiency of 97.8% and low intra- and inter-assay variabilities. The 16S rRNA positivity rate among RLEP positive patients was 66.7% (10/15). Among the five patients without detectable viable *M. leprae*, one was probably subject to sampling error as GAPDH mRNA RT qPCR was also negative.

The present study constitutes the first application of an RNA based viability assay for *M. leprae* on nasal swabs. The RNA assay in combination with quantification by RLEP qPCR was successfully tested for monitoring the treatment response in two MB patients from Germany. Clinical samples of the Togolese validation cohort were not bacteriologically confirmed as neither microscopic assessment of skin smears nor histopathological analysis of skin biopsies was part of the routine diagnostic procedure in Togo. However, for one of our German patients we had the opportunity to extensively analyze corresponding nasal swab samples by microscopy and the two molecular assays. A correlation was found between the bacillary load as determined by RLEP qPCR and BI, as well as the presence of viable bacilli as detected by the RNA assay and MI [38]. It must be noted that the viability assay developed by our group does not allow quantification of RNA as gene expression studies were out of the scope of this work.

Meanwhile investigations on human-to-human transmission and nasal carriage of viable *M. leprae* among untreated MB patients and contact persons in Togo are underway [unpublished data]. Whereas the application of the molecular assays presented no challenges in our German laboratory, several limitations may hamper the realization of its full potential in resource poor settings. The assays in their current format are costly and need adequate infrastructure. Some reagents require expensive dry-ice shipping and continuous stocking of the reagents in Togo means at least three shipments per year. This in turn implies the need for a well-established laboratory management system, which in Togo has been provided by the accredited laboratory of INH [39, 40]. Whereas in Germany the follow-up of leprosy patients can be done in close intervals as needed, in endemic regions beside test costs also the significant mobility of populations in endemic regions may impede close routine follow-up.

## Conclusions

In order to provide laboratory based management and follow-up of leprosy patients attending our outpatient clinic in Munich, Germany, as well as to facilitate transmission studies in Africa, a combined RLEP/16S rRNA (RT) qPCR assay was developed. The assay provides a sensitive and specific tool to determine the bacterial load and viability of *M. leprae* from nasal swab samples and is applicable for early diagnosis, monitoring treatment response and investigating the role of nasal carriage of *M. leprae* in human-to-human transmission through aerosol infection. Whereas in our own setting fortunately neither financial nor logistical restraints hampered the application of the assay on individual patients, for the most part these assays may not be applicable for individualized medicine but rather for epidemiological research issues.

## Additional file

Additional file 1: The additional file comprises: Protocol 1. Conventional extraction of *M. leprae* DNA from clinical specimens; pp. 1–4. Protocol 2. RLEP PCR run protocol; pp. 5–6. Protocol 3. RLEP qPCR run protocol; pp. 7–8. Protocol 4. 16S rRNA RT qPCR run protocol; pp. 9–10. Protocol 5. Preparation of PANTA transport medium and stabilization of *Mycobacterium leprae* DNA/RNA in swab samples; pp. 11–13. Protocol 6. FastPrep extraction of *M. leprae* DNA from clinical specimens; pp. 14–17. Protocol 8. GAPDH mRNA (RT) qPCR run protocol; pp. 26–27. **Table S1.** Inter-assay variability; pp. 28. **Table S2.** Intra-assay variability; pp. 29. (PDF 265 kb)

## Data Availability

All data generated and analyzed during the current study are included in this published article and its supplementary information files. Raw datasets used and analyzed during the current study are available from the corresponding author on reasonable request.

## Abbreviations

6-FAM: 6-Carboxyfluorescein
95%-CI: 95% confidence interval
b: Crossing point of the standard curve with the y-axis
BBQ: BlackBerry quencher
BI: Bacteriological index
BL: Bacillary load
CLS: Cell lysis solution
CN: Copy number
CNV: Copy number variation assay
Ct: Cross threshold
DAHW-T: German Leprosy and Tuberculosis Relief Association, Togo office
DITM: Department of Infectious Diseases and Tropical Medicine
E: Efficiency
GAPDH: Glyceraldehyde-3-phosphate-dehydrogenase
IC: Informed consent
INH: Institut National d’Hygiène
LMU: Medical Center of the University of Munich, Ludwig-Maximilians University
LOD: Limit of detection
MB: Multibacillary
MDT: Multidrug therapy
MI: Morphological index
PANTA: Transport medium (comprising Polymyxin B, Amphotericin B, Nalidixic acid, Trimethoprim, Azlocillin)
PB: paucibacillary
PNLUB-LP: Programme National de Lutte contre l’Ulcère de Buruli, la Lèpre et le Pian
qPCR: quantitative polymerase chain reaction
RIF: Rifampin
RLEP: *Mycobacterium leprae* repeated element
SQ: Starting quantity
SSS: Slit skin smears
TLS: Tissue lysis buffer
y: Slope of the regression line
