# Supplementary material for: Development of a combined RLEP/16S rRNA (RT) qPCR assay for the detection of viable M. leprae from nasal swab samples
Source: BMC Infect Dis. 2019 Aug 28;19:753. doi: 10.1186/s12879-019-4349-9 (PMC6714382; doi:10.1186/s12879-019-4349-9)
Supplement: Supplementary file 1 — The additional file comprises: Protocol 1. Conventional extraction of M. leprae DNA from clinical specimens; pp. 1–4. Protocol 2. RLEP PCR run protocol; pp. 5–6. Protocol 3. RLEP qPCR run protocol; pp. 7–8. Protocol 4. 16S rRNA RT qPCR run protocol; pp. 9–10. Protocol 5. Preparation of PANTA transport medium and stabilization of Mycobacterium leprae DNA/RNA in swab samples; pp. 11–13. Protocol 6. FastPrep extraction of M. leprae DNA from clinical specimens; pp. 14–17. Protocol 8. GAPDH mRNA (RT) qPCR run protocol; pp. 26–27. Table S1. Inter-assay variability; pp. 28. Table S2. Intra-assay variability; pp. 29. (PDF 265 kb) [file 12879_2019_4349_MOESM1_ESM.pdf]

Development of a Combined RLEP/16S rRNA (RT) qPCR Assay for the Detection of Viable *M. leprae* from Nasal Swab Samples.

Marcus Beissner, Anna Wöstemeier, Malkin Saar, Kossi Badziklou, Issaka Maman, Charlotte Amedifou, Magdalena Wagner, Franz X Wiedemann, Komi Amekuse, Basile Kobara, Karl-Heinz Herbinger, Abiba Banla Kere, Thomas Löscher, and Gisela Bretzel.

Additional file - Protocol 1

Conventional extraction of *M. leprae* DNA from clinical specimens

DITM (LMU), INH, DAHW-T **v1 26.05.2012**

1. General considerations

▲ = Note !

This document describes the preparation of *M. leprae* genomic DNA (gDNA) from clinical swab samples.

DNA extraction must be performed on a "DNA extraction bench". The work space must strictly be used for this purpose only, free of DNA amplicons.

▲ ***The laboratory is equipped with all necessary lab equipment (i.e. set of pipettes, pipette filter tips, gloves and lab coats) that are exclusively used in this room.***

2. DNA Extraction

For DNA extraction procedures the Gentra Puregene method (Qiagen, Hilden, Germany) is used with minor modifications of the manufacturer's protocol as described below.

2.1 Reagents

- Cell Lysis Solution, e.g. 1000 ml (CLS, ref# 158908, Qiagen)
- DNA Hydration Solution, e.g. 500 ml (DNA Hyd., ref# 158916, Qiagen)
- Protein Precipitation Solution, e.g. 350 ml (PPS, ref# 158912, Qiagen)
- Lysozyme (10 mg/ml), e.g. 10g (ref# A4972,0010, Applichem, Darmstadt, Germany)
- Proteinase K (20 mg/ml), e.g. 5 ml (ref# 158920, Qiagen)
- Ethanol 70%, e.g. 1L (e.g. ref# A0913,1000, Applichem)
- 2-Propanol (Isopropanol), e.g. 1L (ref# A3465,1000, Applichem)
-

2.2 Materials and instruments

- Lab coats
- Lab gloves (disposable, non sterile)

- Pipettes (0,1-10 µl, 2-20 µl, 20-100 µl, 100-1000 µl)
- DNase-free Pipette filter tips (10 µl, 20 µl, 100 µl, 1000 µl; e.g. Kisker, Steinfurt, Germany)
- 2 ml RNAse/DNAse free screw cap tubes (ref# 72.694.100 Sarstedt, Nümbrecht, Germany)
- DNase-free reaction tubes, 1,5 ml and 2 ml (e.g. Kisker)
- Ice bath
- Table centrifuge, Themomixer, Vortex

2.3 Storage and preparation of reagents

▲***All reagents are checked for their date of expiry before use!***

▲***Thaw frozen reagents completely to room temperature before use!***

▲***Ambient Temperature (AT) is defined as 14-25°C!***

To avoid the contamination risk all reagents must be aliquoted in single-use-volumes and stored as described below:

- CLS buffer is used as transport- and storage medium for diagnostic PCR samples. It is aliquoted by 700 µl and stored at AT until expiry date.
- PPS-solution and DNA Hyd. are aliquoted according to the amount of samples subjected to DNA extraction and stored at AT until expiry date.
- Isopropanol is aliquoted to 700 µl and stored at AT in the dark until expiry date.
- Ethanol (70%) is stored at AT in the dark until expiry date and filled from the bottle to a 50 ml falcon tube before use (if ethanol needs to be diluted from 100% to 70%: 35ml of 100% Ethanol is added to 15 ml distilled water directly before use). Any remaining ethanol in the 50 ml falcon tube is discarded directly after use.
- Lysozyme 10 mg/ml is aliquoted by 100 µl, aliquots are stored at –20° up to 1 year.
- Proteinase K 20 mg/ml is aliquoted by 100 µl, aliquots are stored at –20° until expiry date.

2.4 Performance of DNA Extraction

Using the Qiagen Puregene method, gDNA can be extracted from nasal swab samples.

The extracted DNA is suitable for further downstream applications (e.g. PCR, LAMP, sequencing etc.).

2.4.1 Prearrangements

Keep clinical samples in CLS always in an upright position!

The samples must all be labelled with patient/sample ID and/or laboratory numbers to ensure the correct allocation of patients and samples.

Upon arrival of samples at the laboratory, specimens are heat-inactivated by incubation at 95°C in the thermomixer for 15 minutes. If the extraction procedure is not carried out on the same day, store samples in the fridge at 4°C for up to 1 month or freeze samples at -20°C for long-term storage.

▲ ***Specimens are not infectious anymore but nevertheless should be treated as "possibly infectious" agents and handled with care! Do not remove the swab stick prior to step 6!***

Before starting the extraction procedure, prepare one empty reaction tube as negative extraction control: Add 700 µl CLS buffer and label the tube as "ExCo" and the date of extraction. The extraction control will be treated the same way as the clinical samples.

2.4.2 DNA extraction from clinical swab samples

Cell lysis

1. Add **15 µl Lysozyme [10 mg/ml]** to each sample and incubate at 37°C in the thermomixer for 1 hour. Let the thermomixer shake the samples gently while incubating.
2. Add **10 µl Proteinase K [20 mg/ml]** to each sample, incubate samples at 55°C in the thermomixer for 4 hours (or max. overnight) until complete lysis. Let the thermomixer shake the samples gently while incubating. Lysozyme is inactivated by Proteinase K.
3. The Proteinase K is inactivated at 95°C for 10 minutes in the thermomixer.

Protein Precipitation

4. Place samples in an ice bath for 5 minutes.
5. Add 230 µl Protein Precipitation Solution (PPS)
6. Vortex vigorously at high speed for 20 seconds to mix PPS uniformly with cell lysate (in CLS).
7. Place the samples in an ice bath for 5 minutes.
8. Centrifuge at 13.000 rounds per minute (rpm) for 5 minutes. The precipitated proteins will form a tight pellet. Remove swab stick carefully and discard.

   If the protein pellet is not tight, repeat step 6, followed by a repeated incubation on ice for 5 minutes, then repeat step 8.

   During centrifugation prepare the respective number of new 2 ml reaction tubes containing 700 µl Isopropopanol (see next paragraph, "DNA Precipitation", step 9).

<u>DNA Precipitation</u>

9. Pour the supernatant containing DNA into a new 2 ml reaction tube containing 700µl 100% Isopropanol (leaving behind the precipitated protein pellet).
10. Mix by inverting tubes gently for 10 times.
11. Centrifuge at 13.000 rpm for 5 minutes.
12. Pour off the supernatant. Add 700 µl 70% Ethanol and invert tubes to wash DNA pellets.
13. Centrifuge at 13.000 rpm for 5 minutes. Carefully pour off the ethanol.

▲ *The pellet may be loose so pour slowly and watch the pellet.*

14. Invert and drain tubes on a clean absorbent paper towel (each tube at a different spot) and allow tubes to air dry at AT or in the thermomixer at 65°C until tubes are completely dry (20 min.). The ethanol must be completely evaporated as this may inhibit PCR.

<u>DNA Hydration</u>

15. Add **100 µl** DNA Hydration Solution (DNA Hyd.).
16. Rehydrate the DNA by carefully pipetting up- and down for 20 times.
17. Incubate eluted DNA in the thermomixer for 30 minutes at 65°C.
18. **<u>Optional:</u>** Measure quality and quantity of extracted whole-genome DNA in a photometer.

3. Storage of DNA Extracts

DNA extracts may be stored at 4°C until further processing for up to 1 week. For long-term storage DNA extracts are stored at –20°C.

Avoid repeated freezing and thawing of the samples as this may lead to degradation of DNA.

Development of a Combined RLEP/16S rRNA (RT) qPCR Assay for the Detection of Viable *M. leprae* from Nasal Swab Samples.

Marcus Beissner, Anna Wöstemeier, Malkin Saar, Kossi Badziklou, Issaka Maman, Charlotte Amedifou, Magdalena Wagner, Franz X Wiedemann, Komi Amekuse, Basile Kobara, Karl-Heinz Herbinger, Abiba Banla Kere, Thomas Löscher, and Gisela Bretzel.

Additional file - Protocol 2: RLEP PCR run protocol

**Assay:**	**RLEP**
**Analysis:**	**Amplification of RLEP for subsequent direct DNA sequencing**
**Type of PCR:**	**conventional PCR**

**Primer: (10µM)**	
**RL-F2**	5' ACCTGATGTTATCCCTTGCAC 3'
**RL-R2**	5' CGCTAGAAGGTTGCCGTATG 3'

	**Number of samples:**		plus 10%:	
**Reagent**	**Single reaction [µl]**	**Mastermix [µl]**	**Concentration of solution**	**Final concentration**
DEPC Water (Carl Roth)	10,30			
RL-F2(TibMolBiol – HPLC)	1,00		10µM	0,5µM
RL-R2 (TibMolBiol – HPLC)	1,00		10µM	0,5µM
PCR Puffer II (Applied Biosystems)	2,00		10x	1x
$MgCl_2$ (Applied Biosystems)	2,00		25mM	2,5mM
dNTP´s (Life Technologies)	1,60		10mM	0,8mM
AmpliTaqGold (Applied Biosystems)	0,10		5 U/µl	0,025 U/µl
Total vol. Mastermix [µl]	18,00			
Template	2,00			
Final volume	20,00			

Step:	Temperature:	Time:	
1	95°C	5min	
2	95°C	15 s	
3	55,5°C	20 s	
4	72°C	16 s	(Step 2-4: 35x)
5	72°C	5min	
6	8°C	hold	Duration: 1:25h

Amplicon detection on 1,25% Agarose - Gel (TAE-light [Carl Roth, Karlsruhe, Germany] + 0,01% GelRed [Biotium, Hayward, USA])

Separate and purify PCR product (214 bp) by Ultrafree_DA Kit (Carl Roth) according to manufacturer's specification

Use purified PCR product for direct DNA sequencing with primer RL-F2 according to the protocol described by Beissner et al. (23)

**Sample number**	**Sample (Patient/ID)**	**Result**	**Dilution**
1			10^
2			10^
3			10^
4			10^
5			10^
6			10^
7			10^
8			10^
9			10^
10			10^
11			10^
12			10^
13			10^
14			10^
15			10^
16			10^
17			10^
18			10^
19			10^
20			10^
21			10^
22			10^
23			10^
24			10^
25			10^
26			10^
27			10^
28			10^
29			10^
30			10^
31			10^
32			10^
33			10^
34			10^
35			10^
36			10^
37			10^
38			10^
39			10^
40			10^
41			10^
42			10^
43			10^
44			10^
45			10^
46			10^
47			10^
48			10^

Remarks: ____

Development of a Combined RLEP/16S rRNA (RT) qPCR Assay for the Detection of Viable *M. leprae* from Nasal Swab Samples.

Marcus Beissner, Anna Wöstemeier, Malkin Saar, Kossi Badziklou, Issaka Maman, Charlotte Amedifou, Magdalena Wagner, Franz X Wiedemann, Komi Amekuse, Basile Kobara, Karl-Heinz Herbinger, Abiba Banla Kere, Thomas Löscher, and Gisela Bretzel.

Additional file - Protocol 3: RLEP qPCR run protocol

Assay: RLEP qPCR
Analysis: Detection of *Mycobacterium leprae* DNA (RLEP)
Type of PCR: Quantitative real-time PCR

**Primer:**(10µM)		**Probe:**(5µM)	
**RLEP-F**	5' gCAgTATCgTgTTAgTgAA 3'	**RLEP-P**	5` 6FAM -CgC CgA Cgg CCg gAT CAT CgA- BBQ 3`
**RLEP-R**	5' CgCTAgAAggTTgCCgTATg3'		

	**Number of samples:**		**plus 10%:**		
**Reagent**	**Single reaction [µl]**	**Mastermix [µl]**	**Concentration of solution**	**Final concentration**	**Remarks**
DEPC Water	8,60				Carl Roth
RLEP-F	1,00		10µM	0,5µM	TibMolBiol - HPLC
RLEP-R	1,00		10µM	0,5µM	TibMolBiol - HPLC
RLEP-P	1,00		5µM	0.25µM	TibMolBiol - HPLC
qPCR Mix Plus	4,00		5 U/µl	1 U/µl	Solis BioDyne
Exo IPC Mix	2,00		10x	1x	Life Technologies
Exo IPC DNA	0,40		50x	1x	Life Technologies
Total vol. Mastermix [µl]	18,00				
Template	2,00				
Final volume	20,00				

Step:	Temperature:	Time:	
1	95°C	15 min.	
2	95°C	15 s	
3	60°C	60 s	(Step 2-3: 40x)

Duration 1:38h

Detection in VIC and FAM channels of the CFX96 real-time PCR detection system (BioRad, Munich, Germany) after each step 3.

Sample number	Sample (Patient/ID)	Result	Dilution
1			10^
2			10^
3			10^
4			10^
5			10^
6			10^
7			10^
8			10^
9			10^
10			10^
11			10^
12			10^
13			10^
14			10^
15			10^
16			10^
17			10^
18			10^
19			10^
20			10^
21			10^
22			10^
23			10^
24			10^
25			10^
26			10^
27			10^
28			10^
29			10^
30			10^
31			10^
32			10^
33			10^
34			10^
35			10^
36			10^
37			10^
38			10^
39			10^
40			10^
41			10^
42			10^
43			10^
44			10^
45			10^
46			10^
47			10^
48			10^

Remarks: ____

Development of a Combined RLEP/16S rRNA (RT) qPCR Assay for the Detection of Viable *M. leprae* from Nasal Swab Samples.

Marcus Beissner, Anna Wöstemeier, Malkin Saar, Kossi Badziklou, Issaka Maman, Charlotte Amedifou, Magdalena Wagner, Franz X Wiedemann, Komi Amekuse, Basile Kobara, Karl-Heinz Herbinger, Abiba Banla Kere, Thomas Löscher, and Gisela Bretzel.

Additional file - Protocol 4: 16S rRNA RT qPCR run protocol

Assay: **16S rRNA RT qPCR**
Analysis: **Detection of *Mycobacterium leprae* cDNA (16S rRNA)**
Type of PCR: **Reverse transcriptase- quantitative real-time PCR**

**Primer:**(10µM)		**Probe:**(5µM)	
**16S TaqF**	5' GCA TGT CTT GTG GTG GAA AGC 3'	**16TP2**	5' 6FAM- CCA TCC TGC ACC GCA AAA A -BBQ 3'
**16S TaqR**	5' CAC CCC ACC AAC AAG CTG AT 3'		

	**Number of samples:**		plus 10%:		
**Reagent**	**Single reaction [µl]**	**Mastermix [µl]**	**Concentration of solution**	**Final concentration**	**Remarks**
DEPC Water	8,60				Carl Roth
16S TaqF	1,00		10µM	0,5µM	TibMolBiol - HPLC
16S TaqR	1,00		10µM	0,5µM	TibMolBiol - HPLC
16S TP2	1,00		5µM	0.2µM	TibMolBiol - HPLC
qPCR Mix Plus	4,00		5 U/µl	1 U/µl	Solis BioDyne
Exo IPC Mix	2,00		10x	1x	Life Technologies
Exo IPC DNA	0,40		50x	1x	Life Technologies
Total vol. Mastermix [µl]	18,00				
Template	2,00				
Final volume	20,00				

Step:	Temperature:	Time:	
1	95°C	15 min.	
2	95°C	15 s	
3	60°C	60 s	(Step 2-3: 40x)

Duration 1:38h

Detection in VIC and FAM channels of the CFX96 real-time PCR detection system (BioRad, Munich, Germany) after each step 3.

**Sample number**	**Sample (Patient/ID)**	**Result**	**Dilution**
1			10^
2			10^
3			10^
4			10^
5			10^
6			10^
7			10^
8			10^
9			10^
10			10^
11			10^
12			10^
13			10^
14			10^
15			10^
16			10^
17			10^
18			10^
19			10^
20			10^
21			10^
22			10^
23			10^
24			10^
25			10^
26			10^
27			10^
28			10^
29			10^
30			10^
31			10^
32			10^
33			10^
34			10^
35			10^
36			10^
37			10^
38			10^
39			10^
40			10^
41			10^
42			10^
43			10^
44			10^
45			10^
46			10^
47			10^
48			10^

Remarks: ____

Development of a Combined RLEP/16S rRNA (RT) qPCR Assay for the Detection of Viable *M. leprae* from Nasal Swab Samples.

Marcus Beissner, Anna Wöstemeier, Malkin Saar, Kossi Badziklou, Issaka Maman, Charlotte Amedifou, Magdalena Wagner, Franz X Wiedemann, Komi Amekuse, Basile Kobara, Karl-Heinz Herbinger, Abiba Banla Kere, Thomas Löscher, and Gisela Bretzel.

Additional file - Protocol 5

Preparation of PANTA transport medium and stabilization of *Mycobacterium leprae* DNA/RNA in swab samples

DITM (LMU), INH, DAHW-T **V1 26.05.2012**

1. General considerations

▲ = Note!

This document describes the standard operating procedure for the preparation of PANTA transport medium and RNA*later* collection tubes to keep *Mycobacterium leprae* viable and *M. leprae* DNA/RNA conserved, respectively, during transport from the field to the laboratory. Furthermore, the stabilization of DNA/RNA from viable mycobacteria in PANTA samples by RNA protect treatment is described. These steps are conducted for subsequent combined whole genome DNA/whole transcriptome RNA extraction from *M. leprae* in clinical samples according to the following procedures:

1) Preparation of PANTA and RNA*later* transport tubes for swab samples

2) Stabilization of *M. ulcerans* from swab samples in PANTA or RNA*later*

2. Reagents, material and instruments

2.1 PANTA transport medium

- Dubos Broth Base, 500g (ref# 238510, BD, Heidelberg, Germany)
- Dubos Medium Albumin, 12x20 ml (ref# 230910, BD)
- BBL MGIT PANTA, 6 pcs. (ref# 245114, BD)
- Glycerol (1,2,3-Propantriol), 99.5% p.a., e.g. 1L (ref# 3783.1, Karl Roth, Karlsruhe, Germany)
- H_2O, molecular grade, e.g. 1 L (ref# T143.3, Carl Roth)
- 2 ml RNAse/DNAse free screw cap tube (ref# 72.694.100, Sarstedt, Nümbrecht, Germany)
- Rack for tubes, sterile 1l glass bottle, bottle top filters, Laminar Flow Cabinet

2.2 RNA later transport medium

- RNA*later* RNA stabilization reagent (ref# 76104, Qiagen, Hilden, Germany)
- 2 ml RNAse/DNAse free screw cap tube (ref# 72.694.100, Sarstedt)
- 1000µl pipette filter tips, DNase/RNase free (e.g. Kisker, Steinfurt, Germany)
- Rack for tubes, 1000 µl pipette, Laminar Flow Cabinet

2.3 Stabilization of mycobacterial RNA/DNA from swab samples in PANTA

- 2 ml tubes with 500 µl PANTA
- RNA Protect Bacteria reagent, 2x100ml (ref# 76506, Qiagen)
- Biosafety cabinet, class II
- Centrifuge, Thermomixer, Freezer (-20°C/-80°C)

3. Preparation of PANTA transport medium

▲ ***Work under sterile conditions in a laminar Flow!***

1) Distil 850 ml water and filter through sterile bottle top filter.

2) Filter glycerol through sterile bottle top filter (this will take a while due to the consistency).

▲ ***Glycerol is heat labile!***

3) Dissolve 1.5 g Dubos Broth Base in 204 ml distilled water and autoclave for 15 min. at 121°C. Allow the solution to cool down to approx. 50°C before adding 12 ml of filtered glycerol and 24 ml Dubos Medium Albumin.

4) Dissolve 2 vials of BBL MGIT PANTA (Polymyxin B, Amphotericin B, Nalidixic acid, Trimethoprim, Azlocillin) in 3 ml filtered distilled water each to obtain PANTA antibiotic mixture.

5) For the preparation of PANTA transport medium add 6 ml of PANTA antibiotic mixture to the autoclaved Dubos Broth Base/glycerol mixture under sterile conditions.

6) To check sterility add 1 ml PANTA transport medium under sterile conditions to two 2 ml screw cap tubes each and incubate at 36°C for 24h (tube 1) and 1 week (tube 2), respectively. A clear medium indicates sterility while a turbid medium indicates contamination with bacteria (tube 1) and/or fungi (tube 2). In case of contamination discard the medium and prepare a new batch PANTA transport medium before use.

▲ ***Store 500 µl PANTA transport medium in 2 ml screw cap tubes at 4-8°C for max. 6 months!***

4. Preparation of RNA*later* transport medium

RNA*later* RNA Stabilization Reagent may form a precipitate during storage below room temperature (15–25°C). In this case, re-dissolve the precipitate by heating to 37°C with agitation before use.

▲ ***Work under sterile conditions in a Laminar Flow!***

1) Add 500 µl RNA*later* reagent using RNAse free filter tips with a 1000µl pipette to each 2ml RNAse free screw-cap tube under sterile conditions!
2) Store RNA*later* transport tubes at ambient temperature (AT) for up to 6 months (or between 4-8°C for long term storage up to 12 months).

5. Stabilization of swab samples in PANTA/RNA*later* for RNA/DNA extraction

▲ ***The swab samples may be transported at AT for up to seven days! Apply the following conservation steps directly upon arrival of clinical samples in the laboratory! Work in a biosafety cabinet class II!***

All samples in PANTA and RNA*later*, respectively are supposed to be directly subjected to DNA/RNA extraction.

5.1. Stabilization of swab samples in PANTA

1) Add 1 ml RNA Protect Bacteria reagent to each swab sample in 500 µl PANTA transport medium (2:1), vortex vigorously for 30 seconds (sec).
2) Incubate specimens for 5 minutes (min). at AT, vortex each sample every minute for 10 sec.
3) Inactivate samples at 95°C for 5 min., incubate on ice for 5 min.
4) Pellet material by centrifugation at 5000g at AT for 5 min., remove swab stick carefully and discard supernatant, keep pellets on ice and continue DNA/RNA extraction with pellet or freeze pellets at -20°C for up to 3 months (or -80°C for up to 12 months) for DNA/RNA extraction at a later point in time.

5.2. Stabilization of swab samples in RNAlater

1) Upon arrival at the laboratory vortex samples in RNA*later* vigorously for 30 sec.
2) Incubate samples for 12 h or overnight (max. 24h) at 2-8°C.
3) Inactivate samples at 95°C for 5 min.
4) Centrifuge samples at 5000g at ambient temperature for 5 min. to pellet material, remove swab stick carefully and discard supernatant.
5) Keep pellets on ice and continue DNA/RNA extraction with pellet or freeze pellets at -20°C for up to 1 month (or -80°C for 12 months) for subsequent DNA/RNA extraction at a later point in time.

Development of a Combined RLEP/16S rRNA (RT) qPCR Assay for the Detection of Viable *M. leprae* from Nasal Swab Samples.

Marcus Beissner, Anna Wöstemeier, Malkin Saar, Kossi Badziklou, Issaka Maman, Charlotte Amedifou, Magdalena Wagner, Franz X Wiedemann, Komi Amekuse, Basile Kobara, Karl-Heinz Herbinger, Abiba Banla Kere, Thomas Löscher, and Gisela Bretzel.

Additional file - Protocol 6

FastPrep extraction of *M. leprae* DNA from clinical specimens

DITM (LMU), INH, DAHW-T **v1 26.05.2012**

1. General considerations

▲ = Note !

This document describes the preparation of *M. leprae* genomic DNA (gDNA) from clinical swab samples.

DNA extraction must be performed on a "DNA extraction bench". The work space must strictly be used for this purpose only and has to be free of DNA amplicons.

▲ ***The laboratory is equipped with all necessary lab equipment (i.e. set of pipettes, pipette filter tips, gloves and lab coats) that are exclusively used in this room.***

2. DNA Extraction

For DNA extraction procedures the "My Budget DNA extraction kit mini" (Bio-Budget, Krefeld, Germany) is used with minor modifications of the manufacturer's protocol as described below.

2.1 Reagents, laboratory material & instruments

- My Budget DNA extraction kit mini (ref# 55-5000-050, Bio-Budget) contains the following components for 50 extraction reactions:
  - Lysis buffer (TLS)
  - Proteinase K
  - Binding buffer (TBS)
  - Washing buffer 1 (HS)
  - Washing buffer 2 (MS)
  - Elution buffer
  - 1.5 ml reaction tubes (x200)
  - 2.0 ml collection tubes (x50)
  - Spin columns (x50)
- Ethanol 96%, p.a., e.g. 500 ml (ref# A1615,0500GL, Applichem, Darmstadt, Germany)

- DEPC (Diethylpyrocarbonate) treated water, e.g. 1L (ref# T143.3, Carl Roth, Karlsruhe, Germany)

  Alternatively, molecular grade water may be used.

- Gloves (disposable, non sterile)
- 2 ml RNAse/DNAse free screw cap tubes (ref# 72.694.100, Sarstedt, Nümbrecht, Germany)
- 50 ml RNAse/DNAse free falcon tubes (e.g. ref# G027, Kisker, Steinfurt, Germany)
- 20-100 µl and 100-1000 µl pipets
- 100 and 1000 µl pipette filter tips, DNase/RNase free (e.g. Kisker)
- Alarm clock, reaction tube rack
- Mini table centrifuge (up to 10.000 g), Thermoshaker (min. 900 rot/min.) and Vortex

2.2 Storage and preparation of reagents

To decrease the risk of contaminations, all reagents should be aliquoted in single-use-volumes and stored as described below. Avoid repeated thawing and freezing of Proteinase K!

▲ ***Ambient temperature (AT) is defined as 14-25°C!***

- The lysis buffer TLS is used as transport- and storage medium for diagnostic PCR samples. TLS is aliquoted by 400 µl in 2 ml screw-cap tubes and stored at AT for up to 12 months.
- Buffers TBS, HS, MS and EB are stored at AT.
- Ethanol (96%) is stored at room temperature in the dark and filled from the bottle to a 50 ml falcon tube before use.
- Lyophilized Proteinase K is stored at -20°C.

<u>Before using the Bio Budget DNA extraction kit for the first time</u>:

- Add 15 ml Ethanol 96% to Buffer HS
- Add 35 ml Ethanol 96% to Buffer MS
- Dissolve lyophilized Proteinase K in 1.5 ml DEPC treated water, aliquote by 250 µl in 2 ml screw-cap tubes (one aliquot for 10 samples) and store at -20°C for up to 12 months.

2.3 Performance of DNA Extraction

▲ ***All centrifugation steps are conducted at AT!***

The extracted DNA is suitable for further downstream applications (e.g. PCR, LAMP, sequencing etc.)

2.3.1 Prearrangements

Keep clinical samples in TLS always in an upright position!

The samples must all be labelled with patient/sample IDs and/or laboratory numbers to ensure the correct allocation of patients and samples.

Upon arrival of samples at the laboratory, specimens are heat-inactivated by incubation at 95°C in the thermoshaker for 15 minutes (min). If the extraction procedure is not carried out on the same day, store samples in the fridge at 4°C for up to 1 month or freeze samples at -20°C for long-term storage.

▲***Specimens are not infectious anymore but nevertheless should be treated as "possibly infectious" agents and handled with care! Do not remove the swab stick prior to step 6!***

Before starting the extraction procedure, prepare one empty reaction tube as negative extraction control: Add 400 µl TLS buffer and label the tube as "ExCo" and the date of extraction. The extraction control will be treated the same way as the clinical samples.

Thaw one aliquot of Proteinase K per 10 samples (including the negative extraction control!).

Pre-heat Thermoshaker to 50°C.

2.3.2 DNA extraction from swab samples and slit skin smears

1. Add 25 µl **Proteinase K** to each sample and vortex vigorously for 5 seconds (sec).
2. Incubate at 50°C in the thermoshaker at high speed (900-1400 rotations /min) for 30 min.
3. Prepare 1.5 ml reaction tubes (two for each sample) and label them for steps 7 and 16.
4. Put spin columns in 2.0 ml collection tubes (one for each sample) and label spin columns.
5. Centrifuge samples (from step 2) at 10.000 x g for 1 min.
6. Remove swab stick from samples and discard swab stick.
7. Transfer the supernatant into a labelled 1.5 ml reaction tube (from step 3).

▲***When adding buffers to spin columns you must not touch membranes with the pipet filter tip!***

8. Add 400 µl **binding buffer TBS** to each sample and mix carefully to avoid foaming by pipetting up and down 10 times.

9. Transfer mixed samples completely into labelled spin columns (from step 4) and centrifuge at 10.000 x g for 2 min. The DNA binds to the membrane of the spin column.
10. In case the supernatant did not pass completely through the spin column, discard the flow through of respective samples and centrifuge at 10.000 x g for 4 min.
11. Discard flow through and collection tubes and put spin columns (containing DNA) in new 2.0 ml collection tubes.
12. Add 500 µl **washing buffer HS** to each spin column and centrifuge at 10.000 x g for 1 min.
13. Discard the flow through and collection tubes and put spin columns in new 2.0 ml collection tubes.
14. Add 750 µl **washing buffer MS** to each spin column and centrifuge at 10.000 x g for 1 min.
15. Discard flow through and collection tubes, put spin columns in new 2.0 ml collection tubes and centrifuge samples at 10.000 x g for 2 min.
16. Discard flow through and collection tubes and put spin columns in labelled 1.5 ml reaction tubes (from step 3).
17. To elute DNA from the membranes add **100 µl elution buffer** to each spin column and incubate samples for 2 min. at ambient temperature.
18. Centrifuge at 6.000 x g for 1 min., discard spin columns. DNA is now eluted in the flow through.
19. Measure quality and quantity of extracted whole-genome DNA in a photometer (e.g. Bio Photometer plus; Eppendorf, Hamburg, Germany) or a fluorometer (e.g. Qubit; Thermo Fischer Scientific, Munich, Germany).

3. Storage of DNA Extracts

DNA extracts may be stored at 4°C until further processing for up to 1 week. For long-term storage DNA extracts are stored at –20°C.

Avoid repeated freezing and thawing of the samples as this may lead to degradation of DNA.

Development of a Combined RLEP/16S rRNA (RT) qPCR Assay for the Detection of Viable *M. leprae* from Nasal Swab Samples.

Marcus Beissner, Anna Wöstemeier, Malkin Saar, Kossi Badziklou, Issaka Maman, Charlotte Amedifou, Magdalena Wagner, Franz X Wiedemann, Komi Amekuse, Basile Kobara, Karl-Heinz Herbinger, Abiba Banla Kere, Thomas Löscher, and Gisela Bretzel.

Additional file - Protocol 7

Simultaneous DNA/RNA extraction from swab samples and reverse transcription of whole transcriptome RNA from *Mycobacterium leprae*

DITM (LMU), INH, DAHW-T **V1 15.02.2013**

1. General considerations

▲ <u>**= Note!**</u>

This document describes the standard operating procedure for simultaneous extraction of whole genome DNA and whole transcriptome RNA (incl. reverse transcription to cDNA) of *M. leprae* from nasal swab samples in two steps:

1) Simultaneous DNA/RNA extraction of *M. leprae* from nasal swab samples
2) Reverse transcription of *M. leprae* whole transcriptome RNA to cDNA

Follow all general precautions for working with RNA to avoid nucleic acid degradation during extraction and **do not process more than 8 samples** at a time to allow quick procession. All plastic ware (i.e. tubes and pipette filter tips) must be certified DNAse/RNAse free.

▲ **Get attentively familiar with all steps described below before getting started as incubation times for RNA extraction are very short and delays are not tolerable at all due to quick degradation of RNA!**

2. Material, reagents and instruments

2.1 Simultaneous DNA/RNA extraction from M. leprae

- AllPrep DNA/RNA Micro Kit (ref# 80284, Qiagen, Hilden, Germany)
- Cell lysis solution (CLS), 125 ml (ref# 158906, Qiagen)
- Proteinase K (>600 mAU/ml, solution), 2 ml (ref# 19131, Qiagen)
- Lysozyme, 1g (e.g. ref# A4972,0001, AppliChem, Darmstadt, Germany)
- Beta-(2)-Mercaptoethanol, 25 ml (e.g. ref# A1108,0025, AppliChem)
- Ethanol 96%, p.a., 500 ml (e.g. ref# A1615,0500GL, AppliChem)
- RNase Away, 2.5 L (e.g. ref#A998.1, Carl Roth, Darmstadt, Germany)
- DEPC (Diethylpyrocarbonate) treated water, 1L (e.g. ref# T143.3,Carl Roth)
- Reaction tubes, 1.5 ml, DNase/RNase free (e.g. Kisker, Steinfurt, Germany)
- 10 µl, 100 µl, 1000µl pipette filter tips, DNase/RNase free (e.g. Kisker)
- Syringes, sterile, 0.5 ml (e.g. Braun, Melsungen Germany)
- 22 Gauge needles, sterile (e.g. Braun)
- Sharps container
- Disposable gloves, nitrile
- Small ice container, crushed ice
- Tube rack, centrifuge, thermomixer, laminar flow, molecular scales, fume hood, vortex

2.2 Reverse transcription of M. leprae whole transcriptome RNA to cDNA

- QuantiTect Reverse Transcription Kit (50) (ref# 205311, Qiagen)
- Reaction tubes, 0.2 ml, DNase/RNase free (e.g. Kisker)
- Reaction tubes, 1.5 ml, DNase/RNase free (e.g. Kisker)
- 10 µl pipette filter tips, DNase/RNase free (e.g. Kisker)
- 0.5-10 µl Pipette
- 2 Thermomixers (or Thermal Cycler), small ice container, crushed ice

3. Simultaneous DNA/RNA extraction from *M. leprae*

3.1. Preparations

▲All steps are conducted at ambient temperature (AT) if not explicitly indicated otherwise. Work quickly, but carefully! Perform centrifugation at 20 - 25°C, do not cool centrifuge below 20°C! Follow all general precautions for working with RNA! Wear gloves at all times and change frequently! Clean the working space, centrifuge and racks with RNAse Away before getting started!

The AllPrep DNA/RNA Micro Kit contains:

Buffers: RLT Plus, RPE, RW1, AW1, AW2 and EB
Buffer RLT Plus
DNA and RNeasy spin column, collection tubes and 1.5 ml reaction tubes

▲Before using the AllPrep DNA/RNA Micro Kit for the first time:

I) Add Ethanol 96%: 44 ml to Buffer RPE, 25 ml to Buffer AW1 and 30 ml to Buffer AW2.

II) Prepare 30 ml Ethanol 80% by adding 5 ml DEPC treated water to 25 ml Ethanol 96%.

III) Prepare 48 ml Ethanol 70% by adding 13 ml DEPC treated water to 35 ml Ethanol 96%.

IV) Generate 10 mg/ml Lysozyme solution by adding 10 mg Lysozyme per 1 ml DEPC treated/RNAse free water, label aliquots with "L" and the date of preparation; store at -20°C for up to 6 months.

General preparation prior to each extraction:

▲ ***Work under a fume hood wearing nitrile gloves! ß-Mercaptoethanol is a competent RNAse inhibitor preventing RNA degradation during lysis of mycobacterial cell walls and compartments! Inhalation and contact with human skin is toxic!***

▲ ***Turn off Air-Con***

A) Add **3.5µl x n** (n = no. of samples processed) **ß-Mercaptoethanol** (ß-ME) to **350µl x n Buffer RLT Plus**. Buffer RLT Plus containing ß-ME is stable at AT for up to one month.

B) Clean all working surfaces with RNase Away.

C) To prepare RNase-free syringes and needles (according to sample size) pass DEPC treated water through entire syringe and discard DEPC treated water!

D) Pre-label DNA spin column & collection tubes as well as RNeasy spin columns (according to sample size).

E) Prepare crushed ice in an ice box.

3.2 Lysis:

▲ ***Before adding Proteinase K (step 3.2.2) – a very competent RNAse inhibitor – samples (or previously frozen pellets) must remain on ice!***

1) Thaw frozen pellets on ice.
2) Add 100 µl CLS, 20 µl Proteinase K (>600 mAU/ml) and 15 µl Lysozyme solution (10mg/ml) to each pellet on ice, vortex for 15 seconds (sec.).
3) Incubate samples for 15 minutes (min.) at 37°C in thermomixer at 900 rounds per minute (rpm) (high speed).
4) Incubate samples for 45 min. at 55°C in thermomixer at 900 rpm (high speed).
5) Add 350 µl Buffer RLT Plus (containing ß-ME) under fume hood, vortex vigorously for 15 sec.
6) Homogenise lysate by slowly (to avoid foaming!) passing it 5 times through the DEPC-treated 22 Gauge needle with syringe.
7) Transfer homogenized lysate from the syringe directly into pre-labelled AllPrep DNA spin columns in a pre-labelled 2 ml collection tube.
8) Centrifuge samples at 9000 g for 30 sec., check if all liquid has passed through membrane; if not: repeat this step. **Do not** discard flow through containing RNA!
9) Transfer flow through in collection tubes immediately on ice for subsequent RNA purification (3.3). Place AllPrep DNA spin columns in new collection tubes, store at 4 – 8°C (for up to 24h) for subsequent DNA purification (5.2).

3.3 RNA extraction and purification:

▲ ***All centrifugations are conducted at ambient temperature (AT)!***

10) Add 350 µl Ethanol 70% to the flow through in RNA collection tubes from step 9, pipet 5 times slowly up and down to mix.
11) Pipet samples quickly - but carefully - to labelled RNeasy spin columns. Avoid touching the membrane with the pipet filter tip.
12) Centrifuge at 9000g for 15 sec., discard flow through and transfer RNeasy spin columns to new collection tubes.
13) Add 700 µl Buffer RW1 to the RNeasy spin columns.
14) Centrifuge at 9000g for 15sec., discard flow through and transfer RNeasy spin columns to new collection tubes.
15) Add 500 µl Buffer RPE to RNeasy spin columns.

16) Centrifuge at 9000g for 15sec., discard flow through and transfer RNeasy spin columns to new collection tubes.

17) Add 500 µl Ethanol 80% to RNeasy spin columns.

18) Centrifuge at 9000g for 2 min., discard flow through with collection tubes.

19) Place spin columns in new collection tubes, open lid and centrifuge at 9000g for 5 min.

20) Discard collection tubes with flow through.

21) Place spin columns in pre-labelled 1.5ml Eppendorf tubes and pipett 25 µl RNAse-free water directly onto the spin column membrane. Avoid touching the membrane with the filter tip!

22) Centrifuge at 9000g for 1 min. to elute RNA. Place eluates immediately on ice!

23) Repeat step No. 21 for a second elution by pipetting the 25 µl RNA eluate obtained in step 22 once more on the spin column membrane to concentrate whole transcriptome RNA during this second elution step. The final volume should reach 25µl. Place RNA eluates immediately on ice or freeze at ≤ -70°C.

4. Reverse transcription of *M. leprae* whole transcriptome RNA to cDNA

4.1. Kit

<u>QuantiTect Reverse Transcription Kit contains:</u>

RT Primer Mix, RT Buffer, RNAse free water, gDNA Wipeout Buffer, Reverse Transcriptase (RT)

4.2. Preparations

I) Premix RT Primer Mix and RT Buffer in a 1:4 ratio (i.e. n x 1 µl RT Primer Mix plus 4 µl x n RT Buffer; n = No. of reactions) and label with RT reaction buffer (stable at -20°C for 12 months).

II) In case the thermomixer does not provide an adapter for 0.2 ml tubes: Prepare 1.5 ml reaction tubes filled with RNAse free water (DEPC treated water) according to the sample size and incubate in thermomixer(s) at 42°C (and 95°C) as adapters.

III) Label 0.2 ml RNAse free PCR reaction tubes.

4.3. Reverse transcription of M. leprae whole transcriptome RNA to cDNA

▲ ***Work on ice!***

▲ ***Incubations may be performed in a thermal cycler or in thermomixers!***

Genomic DNA wipeout (gDNA elimination):

24) Add 2 µl gDNA Wipeout Buffer to 12 µl template RNA from step 23 in pre-labelled 0.2 ml reaction tubes. Mix by flicking tubes, centrifuge briefly to ensure that all potentially contaminating DNA is located in the reaction mix!

25) Store remaining RNA on ice for subsequent (optional) quantification or gel-electrophoresis for quality control purposes or freeze at ≤ -70°C in 200µl Ethanol 96% per sample for conservation as RNA backup samples.

26) Incubate samples at 42°C for 5 min.

27) Inactivate reactions at 95°C for 3 min. and put samples immediately on ice.

28) Aliquot 1 wipeout control (2 µl) per sample (wipeout control = genomic DNA wipeout without subsequent reverse transcription) as negative control and store wipeout control aliquots and remaining gDNA eliminated RNA samples on ice. The gDNA eliminated RNA samples will be subsequently subjected to reverse transcription in step 29!

Reverse transcription:

▲ ***Keep RT always on ice. The enzyme remains liquid in frozen state of aggregation!***

29) Add 1 µl RT, 5 µl premixed RT reaction buffer (containing random hexamer primers) and 2 µl RNAse free water (provided in the kit) to gDNA eliminated RNA samples from step 28. Incubate at 42°C for 15 min.

30) Inactivate RT at 95°C for 3 min. and store transcribed cDNA samples at -20°C for long-term storage (until shipment and/or further processing by real-time qPCR).

▲ ***The complementary DNA (cDNA) obtained is single stranded and therefore stable for several years at -20°C but only for at least 4 weeks at 4-8°C and 7-10 days at AT.***

5. DNA extraction

▲ ***Reagents and material as described under 2.1 & 2.2. Samples from step 3.2.9 are processed!***

5.1 Preparations:

I) Preheat Buffer EB in a single aliquot to 70°C (50 µl + 10 % per sample) prior to DNA elution.

5.2 DNA extraction and purification:

31) Add 500µl Buffer AW1 to labelled AllPrep DNA spin columns from step 9 (3.2).
32) Centrifuge samples at 9000g for 15sec., discard flow through.
33) Add 500µl Buffer AW2 to each spin column.
34) Centrifuge samples at 9000g for 2 min., discard collection tube with flow through. If column contacts flow through: repeat step 31) and centrifuge again at 9000g for **1 min.**! Afterwards proceed until step 39).
35) Place spin columns in pre-labelled 1.5ml reaction tubes and add 50 µl pre-heated (70°C) Buffer EB (from step 5.1.I) directly onto spin column membrane without touching the membrane!
36) Incubate samples for 2 min at AT.
37) Centrifuge samples at 9000g for 1 min.
38) Discard AllPrep DNA spin columns - DNA is now in eluates.
39) Store DNA extracts at 4-8°C for short-term (max. 7 days) storage and -20°C for long-term storage.

ANNEX

A.1 Extraction of conserved RNA in Ethanol and Reverse transcription of M. leprae whole transcriptome RNA to cDNA

▲ ***Work on ice!***

▲ ***Incubations may be performed in a thermal cycler or in thermomixers!***

<u>RNA extraction from Ethanol:</u>

i. Centrifuge backup RNA samples in 200 µl Ethanol 96% from step 25 at 13.000 x g for 15 min. at 4°C.

ii. Discard supernatant.

iii. Allow tubes to dry completely in a thermomixer at 95°C for 20 min.

iv. Put samples on ice and add 12 µl RNAse free-water to each sample.

<u>Genomic DNA wipeout (gDNA elimination):</u>

v. Add 2 µl gDNA Wipeout Buffer to 12 µl template RNA from step iv in pre-labelled 0.2 ml reaction tubes. Mix by flicking tubes, centrifuge briefly to ensure that all potentially contaminating DNA is located in the reaction mix!

vi. Incubate samples at 42°C for 5 min.

vii. Inactivate reactions at 95°C for 3 min. and put samples immediately on ice.

viii. Aliquot 1 wipeout control (2 µl) per sample (wipeout control = genomic DNA wipeout without subsequent reverse transcription) as negative control and store wipeout control aliquots and remaining gDNA eliminated RNA samples on ice. The gDNA eliminated RNA samples will be subsequently subjected to reverse transcription in step ix!

<u>Reverse transcription:</u>

▲ ***Keep Reverse Transcriptase always on ice. The enzyme remains liquid in frozen state of aggregation!***

ix. Add 1 µl Reverse Transcriptase, 5 µl premixed RT reaction buffer (containing primers & buffer) and 2 µl RNAse free water (provided in the KIT) to gDNA eliminated RNA samples from step viii. Incubate at 42°C for 15 min.

x. Inactivate Reverse Transcriptase at 95°C for 3 min. and store transcribed cDNA at -20°C for long-term storage.

Development of a Combined RLEP/16S rRNA (RT) qPCR Assay for the Detection of Viable *M. leprae* from Nasal Swab Samples.

Marcus Beissner, Anna Wöstemeier, Malkin Saar, Kossi Badziklou, Issaka Maman, Charlotte Amedifou, Magdalena Wagner, Franz X Wiedemann, Komi Amekuse, Basile Kobara, Karl-Heinz Herbinger, Abiba Banla Kere, Thomas Löscher, and Gisela Bretzel.

Additional file - Protocol 8: GAPDH mRNA (RT) qPCR run protocol

**Assay:**	**GAPDH mRNA RT qPCR**
**Analysis:**	**Detection of GAPDH - mRNA (cDNA)**
**Type of PCR:**	**Reverse transcriptase Quantitative Real-Time PCR**

**Primer:** (10µM)		**Probe:** (5µM)	
**GAPDH fwd**	5' gAAggTgAAggTCggAgTC 3'	**GAPDH TM**	5' 6FAM-CAAgCTTCCCgTTCTCAgCCT--BBQ 3'
**GAPDH rev**	5' gAAgATggTgATgggATTTC 3'		

	**Number of samples:**		plus 10%:		
**Reagent**	**Single reaction [µl]**	**Mastermix [µl]**	**Concentration of solution**	**Final concentration**	**Remarks**
DEPC Water	8,60				Carl Roth
GAPDH fwd	1,00		10µM	0,5µM	TibMolBiol
GAPDH rev	1,00		10µM	0,5µM	TibMolBiol
GAPDH TM	1,00		5µM	0.2µM	TibMolBiol
qPCR Mix Plus	4,00		5 U/µl	1 U/µl	Solis BioDyne
Exo IPC Mix	2,00		10x	1x	Life Science
Exo IPC DNA	0,40		50x	1x	Life Science
Total vol. Mastermix [µl]	18,00				
Template	2,00				
Final volume	20,00				

Step:	Temperature:	Time:	
1	95°C	15 min.	
2	95°C	15 s	
3	60°C	60 s	(Step 2-3: 40x)

Duration 1:38h

Detection in VIC and FAM channels of the CFX96 real-time PCR detection system (BioRad, Munich, Germany) after each step 3.

Sample number	Sample (Patient/ID)	Result	Dilution
1			10^
2			10^
3			10^
4			10^
5			10^
6			10^
7			10^
8			10^
9			10^
10			10^
11			10^
12			10^
13			10^
14			10^
15			10^
16			10^
17			10^
18			10^
19			10^
20			10^
21			10^
22			10^
23			10^
24			10^
25			10^
26			10^
27			10^
28			10^
29			10^
30			10^
31			10^
32			10^
33			10^
34			10^
35			10^
36			10^
37			10^
38			10^
39			10^
40			10^
41			10^
42			10^
43			10^
44			10^
45			10^
46			10^
47			10^
48			10^

Remarks: ____

Additional file – Table 1

Inter-assay variability

qPCR Target	Standard concentration	Ct value day 1[a]	Ct value day 2[a]	Ct value day 3[a]	Ct Std. Dev[b]	CV[c]	Ct range max[d]
RLEP	10^2	30.53	30.62	30.49	0.099	0.324	0.13
	10^3	27.61	27.42	27.34	0.178	0.649	0.25
	10^4	24.45	24.12	24.01	0.222	0.919	0.43
	10^5	20.72	20.52	20.65	0.100	0.485	0.20
	10^6	17.34	17.14	17.01	0.158	0.922	0.33
	10^7	14.27	13.92	13.88	0.211	1.503	0.39
16S rRNA	10^1	35.25	35.00	35.01	0.185	0.527	0.24
	10^2	32.20	32.13	32.17	0.113	0.351	0.04
	10^3	28.82	28.74	28.95	0.080	0.276	0.21
	10^4	25.33	25.35	25.41	0.077	0.304	0.06
	10^5	21.65	21.60	21.54	0.077	0.357	0.13
	10^6	18.17	18.16	18.11	0.086	0.472	0.06
	10^7	14.39	14.34	14.75	0.169	1.167	0.41

The table shows the inter-assay variability of RLEP qPCR and 16S rRNA RT qPCR.

[a]cycle threshold (Ct) values of samples tested in the same dilution

[b]Standard deviation of Ct values

[c]Coefficient of variation (CV) of copy numbers from samples tested in the same dilution

[d]Maximum Ct variation of all samples tested on three different days

Additional file – Table 2

Intra-assay variability

qPCR Target	Standard concentration	Ct value run No. 1[a]	Ct value run No. 2[a]	Ct value run No. 3[a]	Ct Std. Dev[b]	CV[c]	Ct range max[d]
RLEP	10^2	30.66	30.50	30.42	0.122	0.400	0.240
	10^3	27.76	27.65	27.42	0.173	0.628	0.340
	10^4	24.37	24.51	24.48	0.074	0.301	0.140
	10^5	20.65	20.79	20.72	0.070	0.338	0.140
	10^6	17.38	17.27	17.37	0.061	0.351	0.110
	10^7	14.28	14.34	14.20	0.070	0.492	0.140
16S rRNA	10^1	35.05	35.34	35.37	0.177	0.501	0.320
	10^2	32.28	32.19	32.12	0.080	0.249	0.160
	10^3	28.86	28.81	28.79	0.036	0.125	0.070
	10^4	25.22	25.41	25.35	0.097	0.383	0.190
	10^5	21.73	21.62	21.60	0.070	0.323	0.130
	10^6	18.09	10.11	18.30	0.116	0.638	0.210
	10^7	14.35	14.42	14.41	0.038	0.263	0.070

The table shows the intra-assay variability of RLEP qPCR and 16S rRNA RT qPCR.

[a]Cycle threshold (Ct) values of samples tested in the same dilution

[b]Standard deviation of Ct values

[c]Coefficient of variation (CV) of copy numbers from samples tested in the same dilution

[d]Maximum Ct variation of all samples tested within one run
